# STING trafficking as a new dimension of immune signaling

**DOI:** 10.1084/jem.20220990

**Published:** 2023-01-27

**Authors:** Devon Jeltema, Kennady Abbott, Nan Yan

**Affiliations:** 1 Department of Immunology, UT Southwestern Medical Center, Dallas, TX, USA

## Abstract

The cGAS–STING pathway is an evolutionarily conserved immune signaling pathway critical for microbial defense. Unlike other innate immune pathways that largely rely on stationary cascades of signaling events, STING is highly mobile in the cell. STING is activated on the ER, but only signals after it arrives on the Golgi, and then it is quickly degraded by the lysosome. Each step of STING trafficking through the secretory pathway is regulated by host factors. Homeostatic STING trafficking via COPI-, COPII-, and clathrin-coated vesicles is important for maintaining baseline tissue and cellular immunity. Aberrant vesicular trafficking or lysosomal dysfunction produces an immune signal through STING, which often leads to tissue pathology in mice and humans. Many trafficking-mediated diseases of STING signaling appear to impact the central nervous system, leading to neurodegeneration. Therefore, STING trafficking introduces a new dimension of immune signaling that likely has broad implications in human disease.

## Teaser

STING signaling is at the center of multiple autoinflammatory and neurodegenerative diseases. The authors review how STING trafficking influences signaling, propose a model of tonic STING signaling, and discuss an emerging link between dysregulated STING trafficking and neurodegenerative disease.

## Introduction and evolution of the cyclic GMP-AMP synthase–stimulator of IFN genes (cGAS–STING) signaling pathway

**Figure 1.**
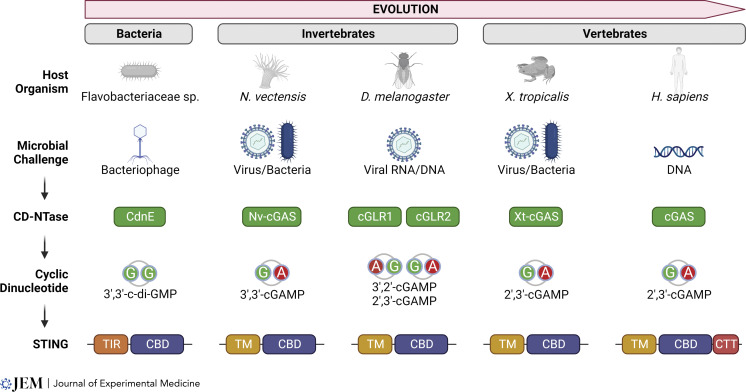
**Evolution of the cGAS–STING pathway.** The cGAS–STING pathway is conserved from bacteria to mammals. The core principle of the CD-NTase→CDN→STING signaling relay is similar. However, each species encodes different CD-NTases that respond to special microbial challenges and then produce distinct CDNs. Each species also encodes STING with different functional domains in addition to the CDN-binding domain that activate different downstream signaling pathways. CBD, cGAMP-binding domain.

A fundamental concept of the innate immune system is the discrimination of “self” versus “non-self.” Several classes of germline-encoded pattern recognition receptors (PRRs), such as TLRs, retinoic acid-inducible gene-I–like receptors (RLRs), and cGAS-like receptors (cGLR), recognize “non-self” components termed pathogen-associated molecular patterns (PAMPs). TLRs and RLRs are highly conserved among divergent animal lineages and are suggested to have evolved more than 600 million yr ago in early metazoan species (Leulier and Lemaitre, 2008). The cGAS–STING pathway is the oldest of the group, with evolutionary history dating much further back to prokaryotes, and its main functionality as a defense system is remarkably conserved from bacteria to mammals (Fig. 1).

Mammalian cGAS senses cytosolic double-stranded DNA and produces the cyclic dinucleotide (CDN) 2′,3′-cyclic GMP–AMP (cGAMP), which binds to and activates STING. STING recruits Tank-binding kinase 1 (TBK1), which phosphorylates STING and itself to recruit interferon regulatory factor 3 (IRF3). Phosphorylation of IRF3 by TBK1 leads to IRF3 translocation to the nucleus to drive the expression of type I IFNs (IFN-I) and IFN-stimulated genes (ISGs; Sun et al., 2013; Wu et al., 2013; Hopfner and Hornung, 2020). The core principle of the cGAS–CDN–STING axis is conserved in bacteria, although upstream triggers and downstream effector responses are divergent. Bacteria produce CDNs to control various cellular processes including antibacteriophage defense systems (Whiteley et al., 2019; Cohen et al., 2019). Notably, the cyclic oligonucleotide-based antiphage signaling system relies on the activation of a CDN synthase (CD-NTase) or a cGLR to produce c-di-GMP, which binds to and activates a bacterial STING homolog. Unlike vertebrate STING, which contains transmembrane domains (TMs), most bacterial and several invertebrate STINGs contain a Toll/interleukin-1 receptor (TIR) domain in place of the TMs. CDN binding to TIR-STING activates the TIR domain, which directs downstream signaling to NAD^+^ hydrolysis or cell death (Morehouse et al., 2020). Furthermore, in contrast to mammals that appear to have a singular cGAS that produces a singular CDN (2′,3′-cGAMP) and one STING, bacteria and flies have multiple cGLRs that can potentially produce multiple types of CDNs to activate distinct types of STING proteins (Cai and Imler, 2021). Although much of the discovery of cGAS and STING homologs is still ongoing, this pathway is almost certainly more versatile than what we know from most studies in mammals.

The presence of STING in eukaryotes enabled the sensing of intracellular bacteria through the detection of CDNs conserved in prokaryotes (Woodward et al., 2010; Dey et al., 2015; Sixt et al., 2017; Tang et al., 2022). The CDN-binding pocket of STING has also evolved from prokaryotes to eukaryotes to preferentially recognize their cognate ligands with higher affinity (Morehouse et al., 2020). Further, the presence of STING in metazoan species accompanied a greater repertoire of effector functions through the addition of a C-terminal tail (CTT). The CTT (aa 341–379 of human STING) is only present in vertebrates, which is crucial for the IFN-I response as it recruits TBK1 and IRF3 (Margolis et al., 2017). Thus, in the long evolutionary history of STING, inducing IFN-I is a very recently acquired function despite being the most widely recognized signaling activity. Other reported signaling functions of STING include NF-κB signaling, autophagy, calcium signaling, unfolded protein response (UPR), cell proliferation, and cellular senescence (Gui et al., 2019; Yamashiro et al., 2020; Wu et al., 2020; Wu et al., 2019; Warner et al., 2017; Cerboni et al., 2017; Moretti et al., 2017; Watson et al., 2015; Yang et al., 2017; Guo et al., 2021; Ishikawa and Barber, 2008; Zhang et al., 2022). Since STING-mediated IFN responses are so strong and prevalent in mammals, IFN-independent activities of STING are often masked in global analyses. Despite this, many IFN-independent activities of STING are beginning to be uncovered (Yamashiro et al., 2020; Wu et al., 2020; Gui et al., 2019; Yum et al., 2021; Ranoa et al., 2019; Wu et al., 2019). These studies collectively suggest that aberrant STING activation in human disease may be further reaching than simply an overproduction of IFN-I.

### Vesicular trafficking in innate immune signaling

Many innate immune PRRs are membrane bound and must be strategically positioned for detecting incoming pathogens. Thus, vesicular trafficking via the endocytosis pathway and the secretory pathway is critical for innate immune signaling. The most well-known examples are TLRs, which are synthesized on the ER and transported via the secretory pathway to their final destinations in the plasma or endosomal membrane. Delayed or defective transport of endosomal TLRs often impairs their maturation and signaling capability (Fitzgerald and Kagan, 2020). Also, the endocytic pathway allows TLR4 to diversify signaling outcomes and C-type lectin receptors to deliver external antigens to the lysosomes (Kagan et al., 2008; Bermejo-Jambrina et al., 2018).

**Figure 2.**
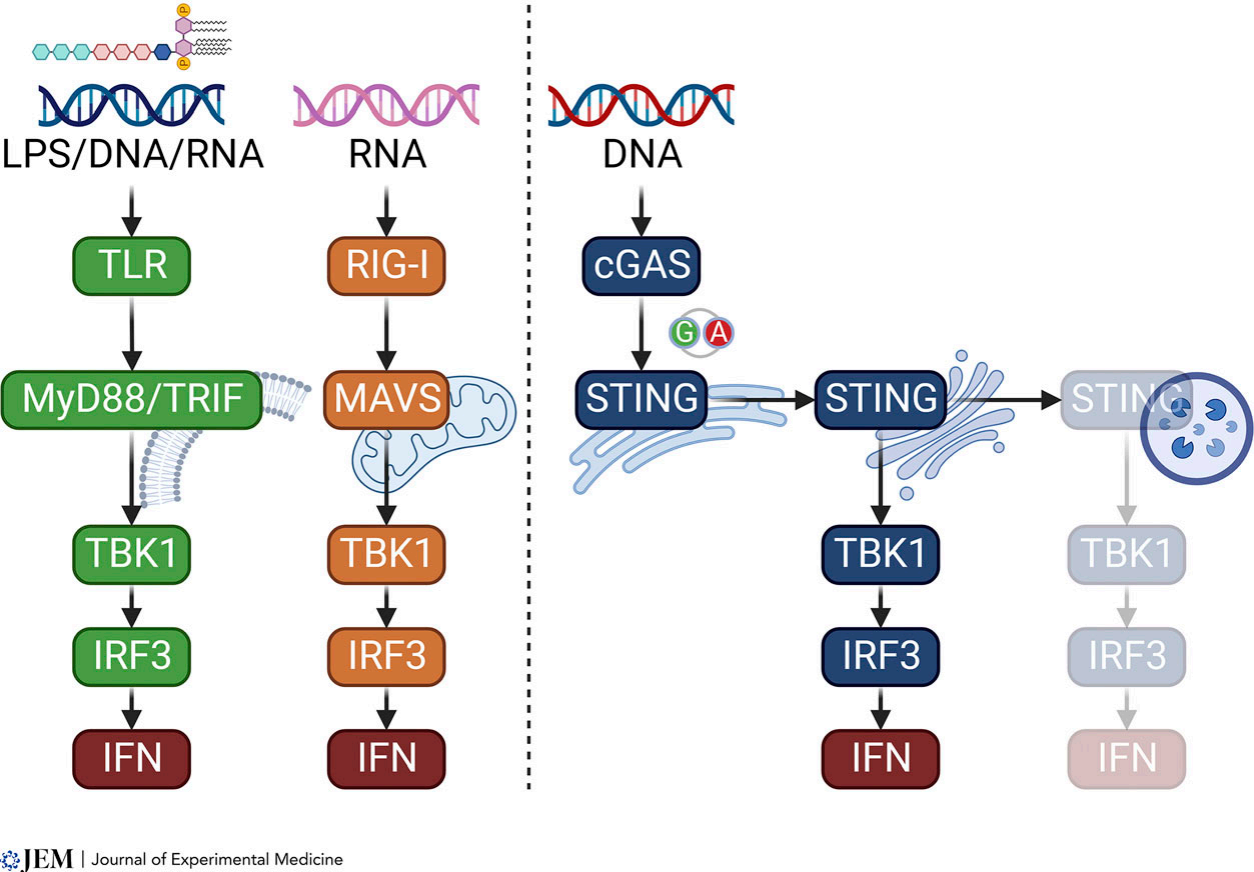
**STING is a unique innate immune adaptor protein that relies on trafficking to signal.** The three major innate immune pathways (e.g., TLR, RIG-I, and cGAS) share a similar signaling cascade. MyD88/TRIF and MAVS assemble signalosomes on the associated membrane and signal in place with no built-in mechanism for turning off the signal. In contrast, STING localizes to the ER but does not signal from the ER. STING signaling turns on then off in a second dimension that involves STING vesicle trafficking from the ER to Golgi and then to the lysosome.

Cytosolic PRRs do not typically rely on vesicular trafficking to either capture PAMPs or propagate downstream signaling. The cGAS–STING pathway is different, in that the signaling outcome is critically regulated by vesicular trafficking. cGAS is present in both the nucleus and cytoplasm where it can sense microbial- or self-DNA and produce cGAMP (Sun et al., 2013; Orzalli et al., 2015). STING is a TM protein on the ER and its activities are dynamically regulated by vesicular trafficking. Interestingly, this trafficking behavior of a membrane-bound innate adaptor protein is unique to STING, as other innate adaptors such as mitochondrial antiviral signaling protein (MAVS), TIR domain–containing adapter-inducing IFN-β (TRIF), and MyD88 do not move within the cell upon activation (Fig. 2).

STING trafficking is conserved across evolution even in the single-cell organism (Woznica et al., 2021). However, it is unclear why STING needs to move and why it cannot just “signal in place” like other innate adaptors do. Is this an ingenious design or evolutionary baggage? What is the fitness benefit of creating this second dimension that would undoubtedly complicate signaling and create more opportunities for microbial antagonism? Indeed, several microbial evasion and immune disorders have been associated with dysregulated STING trafficking (Liu et al., 2014; Dobbs et al., 2015; Deng et al., 2020a; Lepelley et al., 2020; Mukai et al., 2021; Steiner et al., 2022). Microbial antagonism of the cGAS–STING pathway, including STING trafficking, has been reviewed recently (Eaglesham and Kranzusch, 2020; Liu et al., 2022a). We will focus this review on the mechanism of STING trafficking and the pathophysiology of human diseases associated with abnormal STING trafficking.

### Mechanism of STING trafficking

**Figure 3.**
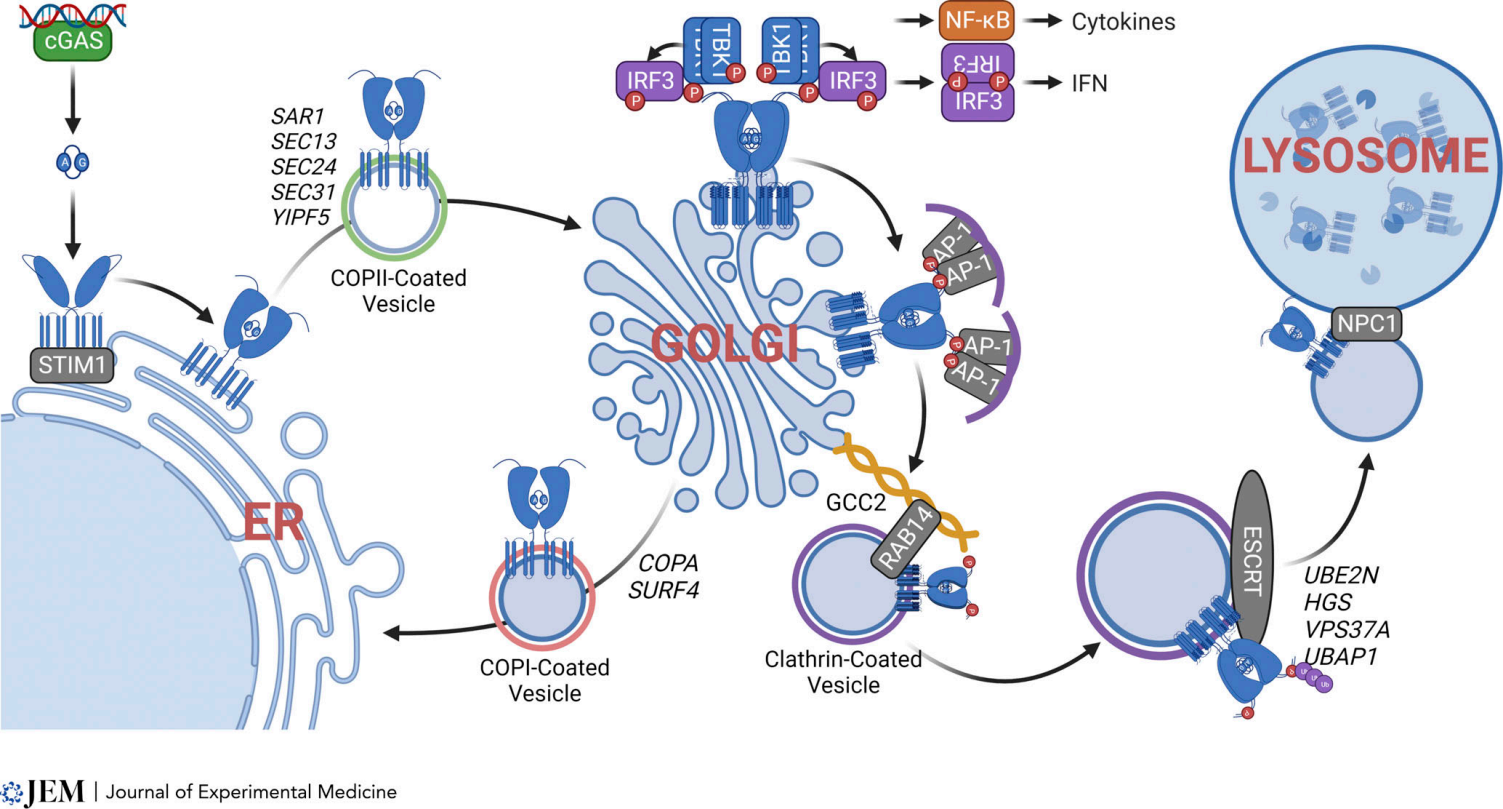
**STING trafficking mechanism.** ER localization of STING at the resting state is actively maintained by ER retention factor STIM1 and COPI vesicle trafficking. CDN-binding triggers STING ER exit and trafficking to the Golgi. STING assembles signalosomes on the TGN and produces immune signaling. Clathrin-associated AP-1 initiates Golgi exit, which also requires Golgin GCC2 and RAB14. Then, STING vesicles are delivered to lysosomes via the ESCRT complex and lysosomal adaptor NPC1. P, phosphorylation.

STING is a TM protein that exists as a dimer on the ER with the CDN-binding domain facing the cytosol. Prior to ligand binding, the linker region of each STING monomer is crossed and the ligand-binding domain is located on the opposite side of the respective transmembrane domain. Binding of cGAMP induces a structural rearrangement in the linker region in which the ligand-binding domain is rotated to be in parallel with the transmembrane domain (Zhang et al., 2013; Gao et al., 2013). This rotation is thought to be critical for STING activation and ER exit via COPII vesicles. STING then translocates to the ER intermediate compartment (ERGIC) and the Golgi where it recruits the kinase TBK1, which phosphorylates STING, itself, and the transcription factor, IRF3. Phosphorylated IRF3 translocates to the nucleus to initiate transcription of IFN-I genes and ISGs. Additionally, STING activates other IFN-independent signaling pathways during this process, although the molecular details are not well defined. STING vesicles then exit the trans-Golgi network (TGN) and continue the journey to endosomes and lysosomes where STING is ultimately degraded to terminate signaling (Fig. 3).

#### Step 1: STING on the ER

STING protein does not contain a signal sequence that would direct it to the ER, raising the question of why STING localizes to the ER and what proteins are responsible for keeping it there. Proteins and processes that help retain STING on the ER include the Ca^2+^ sensor stromal interaction molecule 1 (STIM1) and retrograde COPI vesicle trafficking. STIM1 controls Ca^2+^ entry into the ER through calcium-release activated calcium channels, which are essential in lymphocyte activation and proliferation (Prakriya and Lewis, 2015). STIM1 also moonlights as an ER retention factor for STING through direct binding (Srikanth et al., 2019). Stimulation with 2′,3′-cGAMP disrupts this interaction, thereby allowing STING ER exit. Deficiency of STIM1 releases STING from the ER leading to tonic IFN-I signaling (Srikanth et al., 2019). These observations explain why *STIM1*-deficient patients present with both lymphopenia (due to calcium flux defect in lymphocytes) and autoimmunity (likely due to STING-IFN signaling; Picard et al., 2009). Golgi-to-ER retrograde trafficking also plays an important role in keeping STING on the ER. This was not immediately obvious until the surprising observation from multiple groups that when the COPI pathway is defective, STING accumulates on the Golgi at the resting state (Deng et al., 2020a; Lepelley et al., 2020; Mukai et al., 2021).

As we will elaborate later, STING is not simply anchored on the ER in the resting state; rather, it continuously moves through the secretory pathway. The combination of ER retention factors and retrograde trafficking likely establishes the “appearance” of STING localization on the ER. Further, there are additional cofactors responsible for regulating STING protein level on the ER, including Toll-interacting protein (TOLLIP), E3 ubiquitin ligase tripartite motif protein 30α (TRIM30α), and tyrosine-protein phosphatase non-receptor type 1 and 2 (PTPN1/2), which regulate lysosomal and proteasomal degradation of STING at homeostasis, respectively (Pokatayev et al., 2020; Wang et al., 2015; Xia et al., 2019; Table 1).

**Table 1.** STING trafficking cofactors

Step	Cofactor	STING function	Protein family	Reference
STING on the ER	STIM1	ER retention	Calcium regulator	Srikanth et al., 2019
	COPA	Retrograde trafficking	COPI vesicle	Mukai et al., 2021
	SURF4	Retrograde trafficking	COPI vesicle	Mukai et al., 2021
	TOLLIP	Lysosomal degradation	−	Pokatayev et al., 2020
	TRIM30α	Proteasome degradation	E3 Ligase	Wang et al., 2015
	PTPN1/2	Proteasome degradation	Ubiquitination	Xia et al., 2019
Translocation from ER to Golgi	STEEP	ER exit		Zhang et al., 2020
	SAR1	ER exit	COPII vesicle	Gui et al., 2019; Ran et al., 2019
	SEC13	ER exit	COPII vesicle	Ran et al., 2019
	SEC23	ER exit	COPII vesicle	Ran et al., 2019
	SEC24C	ER exit	COPII vesicle	Gui et al., 2019
	SEC31	ER exit	COPII vesicle	Ran et al., 2019
	YIPF5	ER exit	Yip1 domain	Ran et al., 2019
	TRAPβ	ER exit	Translocon	Ishikawa and Barber, 2008; Luo et al., 2016
	IRHOM2	ER exit	Translocon	Luo et al., 2016
	ATG9A	ER exit	Autophagosome	Saitoh et al., 2009
	SCAP	IRF3 interaction	Lipid trafficking	Chu et al., 2021; Chen et al., 2016
Post-Golgi trafficking and degradation by the lysosome	AP-1	Golgi exit	Adaptor complex	Liu et al., 2022b
	GCC2	Golgi exit	Golgin	Tu et al., 2022
	RAB14	Golgi exit	RAB GTPase	Tu et al., 2022
	UBE2N	Lysosomal degradation	Ubiquitination	Gentili et al., 2022 *Preprint*
	HGS	Lysosomal degradation	ESCRT	Gentili et al., 2022 *Preprint*
	VPS37A	Lysosomal degradation	ESCRT	Gentili et al., 2022 *Preprint*
	UBAP1	Lysosomal degradation	ESCRT	Gentili et al., 2022 *Preprint*
	TSG101	Lysosomal degradation	ESCRT	Kuchitsu et al., 2022 *Preprint*
	VPS4	Lysosomal degradation	ESCRT	Kuchitsu et al., 2022 *Preprint*
	NPC1	Lysosomal degradation	Lipid trafficking	Chu et al., 2021
	C9ORF72	Lysosomal degradation	RAB GTPase regulator	McCauley et al., 2020
	UNC93B1	Lysosomal degradation	−	Zhu et al., 2022; He et al., 2021

This is not a comprehensive list. Only cofactors discussed in this review are shown.

#### Step 2: Translocation from ER to Golgi

After ligand binding, STING translocation to the Golgi is initiated from the ER-exit sites. This process requires COPII vesicle assembly. Loss of Sar1, Sec13, Sec24, and Sec31 prevent STING ER exit upon ligand binding and attenuates STING signaling (Gui et al., 2019; Ogawa et al., 2018; Sun et al., 2018). Deletion of the ERGIC/Golgi protein YIPF5, which facilitates COPII vesicle budding and fusion with targeted membranes, also blocks STING ER exit (Ran et al., 2019). Further, STING ER exit protein (STEEP) stimulates ER membrane curvature by promoting ER PtdIns(3)P accumulation, thereby facilitating COPII vesicle formation and STING ER exit (Zhang et al., 2020).

In addition to the classical COPII vesicle apparatus, several other proteins and complexes on the ER are important for STING ER-to-Golgi transport (Table 1). In the absence of the translocon complex component TRAPβ, ligand-induced STING trafficking is prevented (Ishikawa and Barber, 2008; Luo et al., 2016). The ER protein iRhom2 facilitates TRAPβ and STING interaction. Similar to *TRAPβ* deficiency, the loss of iRhom2 also prevents STING ER exit (Luo et al., 2016). Since TRAPβ and iRhom2 do not affect protein trafficking via the COPII apparatus, it is possible that they regulate an additional step of STING lateral movement on the ER to reach the ER-exit sites.

Once STING reaches the Golgi, a chain of biochemical events is initiated: (1) STING recruits TBK1 via the CTT; (2) TBK1 phosphorylates STING (at serine 366) and itself; (3) STING oligomerizes and recruits IRF3 to the pLxIS motif where TBK1 phosphorylates IRF3; (4) p-IRF3 dimerizes and translocates to the nucleus to turn on immune gene expression. CryoEM structural studies provide clear visual evidence of these processes at the atomic level (Hopfner and Hornung, 2020; Decout et al., 2021; Zhang et al., 2019; Shang et al., 2019; Zhao et al., 2019; Ergun et al., 2019). In addition to phosphorylation, other posttranslational modifications are critical for STING signaling on the Golgi (Yu et al., 2022). For example, STING is palmitoylated at cysteine 88 and 91 by Golgi-localized acyltransferases DHC3/7/15 at cis-Golgi (Mukai et al., 2016). Blocking STING palmitoylation with nitrofuran derivatives, C-178, C-179, and H-151, prevents STING oligomerization and signalosome assembly (Haag et al., 2018). Ubiquitination of STING by multiple proteins (AMFR, INSIG1, ZDHHC1, RNF26, TRIM32, and TRIM56) is also important for signaling on the Golgi (Shu and Wang, 2014). Further, several protein cofactors directly or indirectly facilitate STING signalosome formation on the Golgi. S6K and SREBP cleavage-activated protein (SCAP) facilitate IRF3 recruitment to STING–TBK1 (Wang et al., 2016; Chen et al., 2016). While ATG9a, a core autophagy protein involved in membrane expansion, negatively regulates the formation of the STING–TBK1 complex and downstream IFN signaling (Saitoh et al., 2009), protein phosphatase Mg^2+^/Mn^2+^-dependent 1A and protein phosphatase 6 catalytic subunit inhibit signaling by dephosphorylating STING (Li et al., 2015; Ni et al., 2020).

ER-to-Golgi translocation is also required for STING IFN-independent activities, although the molecular mechanism and chain of events are much less understood. For example, STING-mediated NF-κB signaling requires TBK1 binding to STING C-terminus on the Golgi but does not require STING phosphorylation (Yum et al., 2021; Wu et al., 2020). STING-mediated autophagy requires STING to translocate from the ER to ERGIC, which is a known source of autophagic membrane (Gui et al., 2019). STING-mediated UPR and cell death require STING ER exit (Wu et al., 2019). Interestingly, both STING-mediated UPR and autophagy activities are mapped to the same motif (an α-helix containing amino acid 322–343), suggesting that these two activities may rely on a common process that is yet to be defined. Whether STING ER exit alone (before it reaches ERGIC or Golgi) induces any cellular signaling also remains unclear. One interesting possibility is altered ER calcium release due to STING translocation-induced ER membrane leakage (Wu et al., 2020; Srikanth et al., 2019; Wu et al., 2019). Calcium signaling also impacts functions of lymphocytes more than myeloid cells, which fits the observation that STING drives mostly IFN-independent activities in lymphocytes (Wu et al., 2020).

#### Step 3: Post-Golgi trafficking and degradation by the lysosome

While there are many known STING cofactors that act at the ER-to-Golgi stage, far fewer cofactors are known for post-Golgi trafficking of STING. This is likely because most loss-of-function genetic screens of STING-mediated IFN responses are inherently biased toward “dependency” cofactors (i.e., cofactors required for IFN-I signaling). Post-Golgi trafficking of STING attenuates IFN signaling. Thus, loss-of-function of these cofactors would prolong STING signaling, which is difficult to capture as an increase in IFN response on a single time-point–readout genetic screen. Therefore, a biochemical approach was needed. Several proteomic screens based on spatiotemporal affinity labeling revealed post-Golgi regulators of STING (Gentili et al., 2022 *Preprint*; Tu et al., 2022; Chu et al., 2021). We can now begin to piece together a step-by-step process that transfers STING from the Golgi to lysosomes.

The process of STING degradation begins as soon as STING is phosphorylated on the Golgi. The adaptor protein complex-1 (AP-1) binds a dileucine motif as well as phosphorylated S366 residue in the STING CTT domain. AP-1 then sorts phosphorylated STING into clathrin-coated vesicles for transport to the endo-lysosomes (Liu et al., 2022b). Suppression of AP-1 enhances STING signaling. STING Golgi exit also requires trans-Golgi GRIP and coiled-coil domain containing protein 2 (GCC2/GCC185). GCC2 belongs to the Golgin family of proteins on the TGN that transfer vesicles to RAB GTPases via APs (Muschalik and Munro, 2018). The TGN serves as a major sorting hub where cargo is sorted into various transport carriers for trafficking to distinct compartments of the cell (Di Martino et al., 2019). Interestingly, although Golgins are functionally redundant, only GCC2 is required for STING Golgi exit. Multiple RAB GTPases, such as RAB14, are required for further transport of STING after Golgi exit. RAB14-knockout phenocopies GCC2-knockout with increased STING Golgi dwell time and increased tonic- and ligand-stimulated IFN-I signaling. Further, *Gcc2*^*−/−*^ mice develop autoimmunity that is genetically dependent on STING (Tu et al., 2022).

Once STING reaches the endosome, it is again ubiquitinated by UBE2N, which forms a platform for endosomal sorting complexes required for transport (ESCRT) recruitment. HGS, VPS37A, UBAP1, Tsg101, and Vps4 are components of the ESCRT complex identified as STING cofactors. Loss-of-function of the ESCRT complex impairs STING degradation by the lysosome (Gentili et al., 2022 *Preprint*; Kuchitsu et al., 2022 *Preprint*). Interestingly, a UBAP1 mutant that underlies spastic paraplegia in humans also leads to a STING-dependent IFN-I response (Gentili et al., 2022 *Preprint*). Recruitment of STING to the lysosome is, in part, mediated by lysosomal protein Niemann-Pick C1 (NPC1). NPC1 directly interacts with STING through transmembrane domains on both proteins and recruits STING vesicles to lysosomes. NPC1 deficiency impairs STING degradation and enhances STING-mediated IFN-I signaling (Chu et al., 2021).

C9ORF72 and UNC93B1 also promote lysosomal degradation of STING, although molecular details remain to be determined (McCauley et al., 2020; He et al., 2021; Zhu et al., 2022). STING also drives non-classical autophagy, which may contribute to its own degradation by autophagosomes. It is also unclear whether STING vesicles fuse with or are engulfed by lysosomes and how the membrane topology works for each scenario.

### Homeostatic functions of STING trafficking: The basal flux model

**Figure 4.**
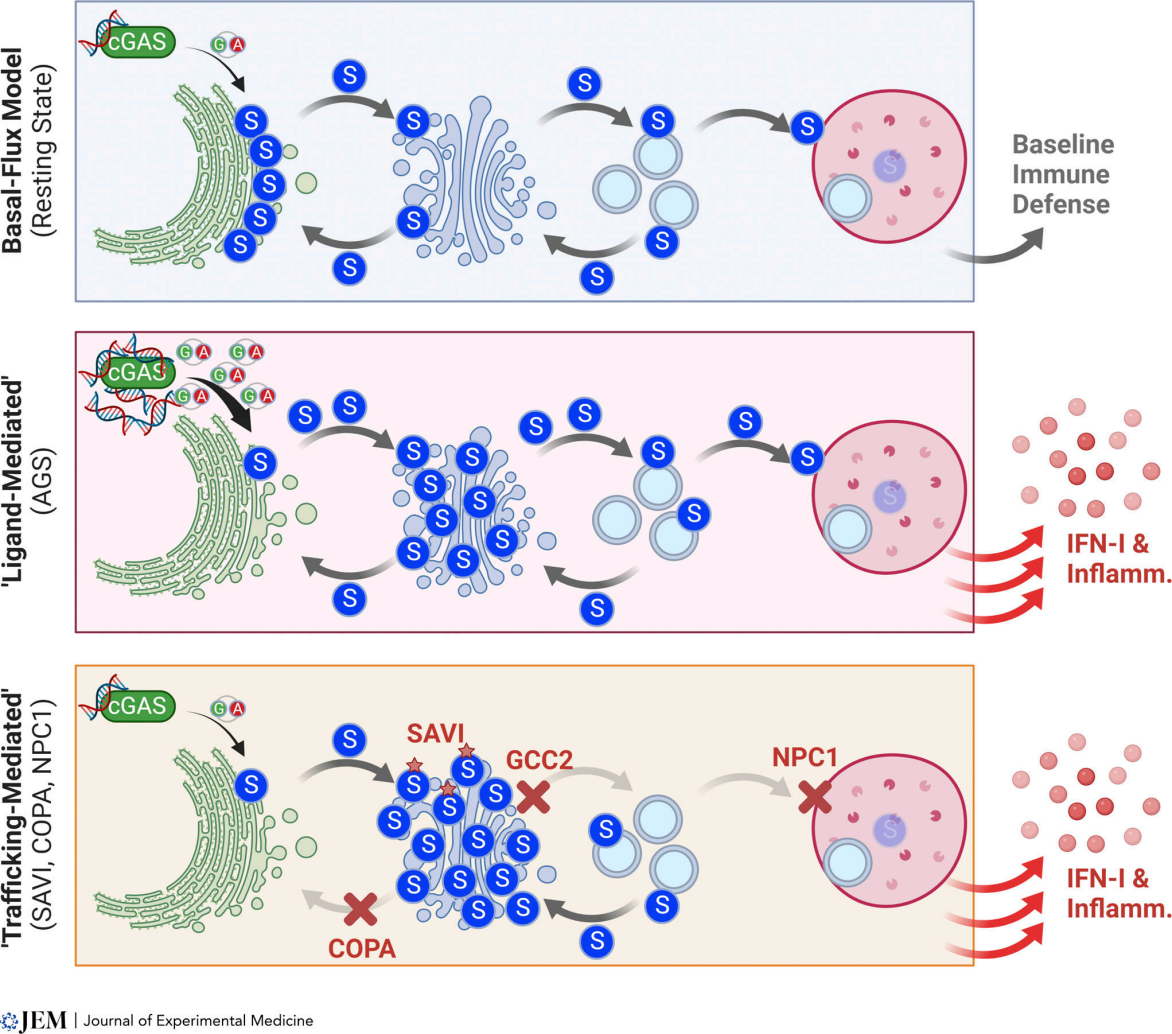
**A basal flux model of the cGAS–STING pathway. **See text for details. S, STING.

Most PRRs are activated by a “trigger release” mechanism where PRRs remain inactive at homeostasis and only signal after encountering their cognate PAMP (Fig. 4). This mechanism explains some antiviral activities of cGAS–STING but fails to explain many other observations. First, *cGas*^*−/−*^ and *Sting*^*−/−*^ cells and mice are more susceptible to a wide range of RNA virus infections although only a few RNA viruses are known to cause the release of pathogenic DNA that activates cGAS (Schoggins et al., 2014; Li et al., 2013; Reinert et al., 2016; Cui et al., 2020; Abe et al., 2013). Similarly, both *cGas*^*−/−*^ and *Sting*^*−/−*^ cells have reduced basal expression of ISGs, which could increase their broad susceptibility to infection (Tu et al., 2022; Schoggins et al., 2014). Second, mutations that perturb STING trafficking alone (in the absence of a ligand) can activate STING signaling, such as gain-of-function mutations seen in STING-associated vasculopathy with onset in infancy (SAVI), loss-of-function mutations seen in COPA syndrome, *GCC2 *deficiency, *AP-1 *deficiency, ESCRT deficiency, and *NPC1 *deficiency (Chu et al., 2021; Lepelley et al., 2020; Tu et al., 2022; Liu et al., 2014; Gentili et al., 2022 *Preprint*; Liu et al., 2022b).

Therefore, a “basal flux” model of the cGAS–STING pathway should be adopted (Fig. 4). Conceptually, this can be appreciated by a “river-dam” analogy. STING continuously flows down a river from the ER to lysosome at homeostasis as opposed to simply being anchored upstream waiting for a ligand to cut the anchor loose. Trafficking interruption is similar to placing a dam in the river to trap STING, which can lead to continuous energy (IFN-I) production even in a slow-moving river.

According to the basal flux model, STING does not remain stationary on the ER. Instead, it continuously moves from the ER to the Golgi. From the Golgi, STING continuously moves back to the ER as well as forward to the lysosome for degradation. Most STING protein in the cell is associated with the ER at any given time, which is observed by microscopy; however, as soon as homeostatic STING trafficking is disturbed (e.g., blocking COPI, Golgi exit, or lysosomal degradation), STING has the potential for spontaneous signaling that can cause tissue damage and disease pathology. How exactly spontaneous STING signaling occurs when trafficking is perturbed is unclear. It is possible that the accumulation of STING on the Golgi is sufficient to spontaneously drive oligomerization and downstream signaling but this requires further exploration.

Another important prediction of the basal flux model is that trafficking-mediated STING signaling is cGAS dependent, as cGAS is required to initiate the flow of STING trafficking and signaling even at homeostasis. cGAS activity is always detectable at homeostasis in the wild-type cell, producing low levels of cGAMP to drive basal STING trafficking and IFN-I signaling (Steiner et al., 2022; Tu et al., 2022). Thus, STING trafficking interruption does not require an additional pathogenic cGAS stimulus to boost IFN-I signaling. Indeed, in a few cases when cGAS was carefully examined, no evidence of nuclear or mitochondrial damage, and no changes in cGAMP levels were observed (Tu et al., 2022). Instead, if cGAS continues producing cGAMP, STING moves along the vesicular trafficking pathway at low levels.

cGAS is not the only driver that can promote STING trafficking. There are at least a couple of examples of cGAS-independent STING trafficking and signaling. In NPC1 disease, STING trafficking is driven by SREBP/SCAP trafficking due to cholesterol depletion on the ER; therefore both STING signaling and NPC1 disease pathology are independent of cGAS (Chu et al., 2021). Gain-of-function STING mutation N154S and V155M can also drive STING trafficking leading to disease pathology independently of cGAS (Dobbs et al., 2015; Luksch et al., 2019). A critical feature of this model is that STING is constantly moving through the vesicular trafficking pathway. In most cases, this will require cGAS, but, in a few cases where an alternative driving mechanism is in place, STING is able to traffic independent of cGAS.

### STING trafficking and disease

#### Ligand-mediated diseases of STING signaling

Diseases caused by aberrant STING activation can be categorized by their initiating factor as either “ligand-mediated” or “trafficking-mediated” (Fig. 4). Ligand-mediated describes diseases that are caused by accumulation of an extracellular or cell-intrinsic DNA trigger, representing the canonical activation of the cGAS–STING pathway. The most common forms of these DNA triggers include extracellular DNA released from dying cells, mitochondrial DNA (mtDNA), and genomic DNA. For example, in Aicardi-Goutières Syndrome (AGS), mutations in cytosolic exonuclease TREX1 lead to an accumulation of self-DNA in the cytosol that is sensed by cGAS to drive STING-dependent IFN-I production leading to a severe autoinflammatory disease (Crow et al., 2006; Gray et al., 2015; Gall et al., 2012; Gao et al., 2015). The presence of a self-DNA trigger is observed in numerous other diseases ranging from polygenic autoimmune diseases to monogenic inborn errors of innate immunity to neurodegenerative diseases such as Parkinson’s disease (PD). These diseases driving canonical cGAS–STING activation are covered extensively in recent reviews (Decout et al., 2021; Crow and Stetson, 2022).

### Trafficking-mediated diseases of STING signaling

**Figure 5.**
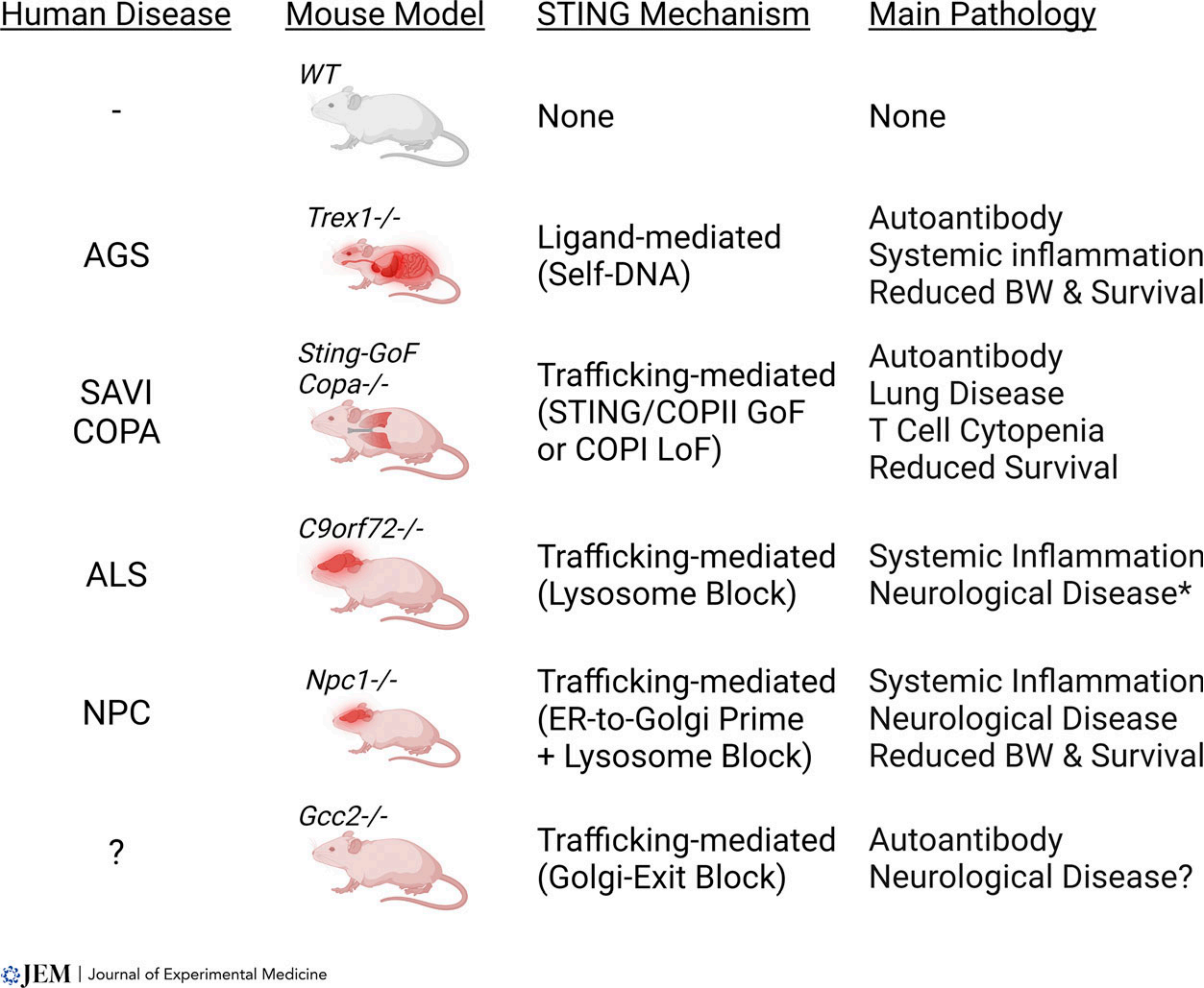
**Trafficking-mediated disease of STING signaling mouse models.** *Trex1*^−/−^ mouse is a model for AGS and ligand-mediated disease of STING signaling. Several recently described mouse models for trafficking-mediated diseases of STING signaling are also shown. COPA, COPA deficiency syndrome. BW, body weight. GoF, gain-of-function. LoF, loss-of-function. Red color indicate tissues affected in the mouse. Asterisk indicates that *C9orf72*^−/−^ mice do not develop ALS, although C9ORF72 is strongly associated with ALS in humans.

Trafficking-mediated describes diseases that occur in the absence of an obvious pathogenic DNA trigger (Fig. 4 and Fig. 5). This does not mean cGAS is not required. As we explained in the previous section, cGAS or an alternative driver of STING trafficking will be required for STING signaling. The first recognized trafficking-mediated disease of STING signaling was identified in 2014 as SAVI (Liu et al., 2014). SAVI is an inherited inflammatory syndrome caused by gain-of-function mutations in *STING1* (Frémond et al., 2021; Bennion et al., 2019). Most cases of SAVI are caused by de novo heterozygous mutations leading to spontaneous polymerization and activation of STING independent of cGAMP binding. Constitutive activation can also be appreciated in the subcellular localization of STING to the ERGIC rather than the ER in SAVI (Ogawa et al., 2018; Mukai et al., 2016). Humans that harbor disease-causing mutations in *STING1* develop early onset interstitial lung disease, cutaneous vasculopathy, and systemic inflammation that result in high mortality rate and severely limited quality of life. Peripheral blood mononuclear cells of SAVI patients show a distinct elevation of IFN-I genes, and SAVI is considered a type I interferonopathy. Despite this, with the exception of a cell-specific knock-in mouse model, the majority of disease pathology in mouse models of SAVI occurs independent of IFN-I (Warner et al., 2017; Luksch et al., 2019; Motwani et al., 2019; Siedel et al., 2020; Bennion et al., 2019; Bouis et al., 2019; Martin et al., 2019). Additionally, inhibition of JAK signaling as a treatment for SAVI is not as effective as in other classical type I interferonopathies such as AGS (Sanchez et al., 2018). A trial of ruxolitinib in eight patients improved overall disease-associated joint and skin manifestations with only limited efficacy on lung disease treated before irreversible damage occurred (Frémond et al., 2021). As such, it is likely that both IFN and IFN-independent activities of STING contribute to SAVI pathogenesis in humans. Of note, N154S and V155M are the two most well-studied SAVI mutations that show clear evidence for homeostatic STING trafficking. Activation mechanisms of more recently reported SAVI mutations remain to be defined.

COPA syndrome represents another example of a trafficking-mediated disease of STING signaling. COPA syndrome is a recently discovered monogenic autoinflammatory disorder characterized by interstitial lung disease, inflammatory arthritis, and high expression of IFN-I and ISGs (Vece et al., 2016). The clinical presentation of COPA syndrome is remarkably similar to SAVI, and mouse models of COPA disease closely recapitulate that found in humans (Frémond and Crow, 2021; Deng et al., 2020b). COPA syndrome is caused by heterozygous mutations in the *COPA* gene, which encodes COP-α of the COPI complex. In *COPA*-mutant patient cells or COPA-deficient mice, STING constitutively localizes to the Golgi leading to chronic IFN-I signaling (Lepelley et al., 2020). Disease in COPA-deficient mice can be rescued by genetic knockout or pharmacological inhibition of STING signaling, establishing STING as a critical driver of COPA syndrome (Deng et al., 2020a). Whether COPA disease phenotypes in mice are IFN-dependent remains to be defined.

Although not yet associated with a known human disease, GCC2 deficiency impairs STING Golgi exit and blocks lysosomal degradation. The overall cellular and tissue phenotypes of GCC2 deficiency are less severe compared with COPA deficiency. This is not surprising as *Gcc2*^*−/−*^ cells display less Golgi localization of STING compared with *Copa*^*−/−*^ cells, which display nearly complete Golgi localization of STING. The tissue pathology seen in *Gcc2*^*−/−*^ mice is also less severe compared with *Copa*^*−/−*^ mice. Both of these phenotypes are cGAS–STING dependent, without evidence of a pathogenic self-DNA trigger (Tu et al., 2022).

Niemann-Pick disease type C (NPC) is another recent example of a trafficking-mediated disease of STING signaling. NPC1 is a lysosomal protein that mediates intracellular cholesterol trafficking and lipid homeostasis. Loss of function mutations in the *NPC1* gene underlie 95% of NPC disease in humans (Touma et al., 2021; Garver et al., 2007). Clinical manifestations of NPC disease include cerebellar ataxia, loss of Purkinje neurons, and progressive cognitive impairment. The biochemical functions of NPC1 in cholesterol trafficking are well understood. We recently identified NPC1 in a proteomic screen as the lysosomal adaptor protein that recruits STING for degradation. In NPC1 deficiency, STING degradation is delayed leading to extended IFN-I signaling. Additionally, NPC1 deficiency leads to cholesterol depletion from the ER, resulting in SREBP2-tethered trafficking of STING from the ER to the Golgi. Importantly, *Sting*^−/−^, but not *cGas*^−/−^, completely rescues neuroinflammation associated with *Npc1*^−/−^ in mice and substantially improves the motor function of these mice (Chu et al., 2021).

### STING trafficking and signaling in neurodegenerative diseases

Diseases driven by excessive STING activation and inflammation often include clinical manifestations of the central nervous system (Table 2). Studies within the last 5 yr have associated STING signaling with a wide range of neurodegenerative diseases including amyotrophic lateral sclerosis (ALS), frontotemporal dementia (FTD), PD, as well as several monogenic diseases such as NGLY1 deficiency (Yang et al., 2018) and NPC disease (Chu et al., 2021). Additionally, recent work has demonstrated neuroinflammation and degeneration of dopaminergic neurons in a mouse model of chronic STING activation (SAVI, N153S; Szego et al., 2022). Both ligand-mediated (e.g., mitochondrial defect) and trafficking-mediated (e.g., endolysosomal defect) STING signaling mechanisms have been implicated in neurodegenerative diseases.

**Table 2.** STING signaling in neurological disease

Neurological disease	Type of evidence for STING involvement	Mode of STING activation	Role of STING signaling
ALS and FTD	Genetic, pharmacological inhibitor	Ligand (mtDNA, e.g., TDP-43), trafficking (e.g., C9ORF72)	Genetic deficiency or inhibition of STING signaling in a mouse model protects against disease progression; STING inhibitor reduces IFN expression in patient cells (Yu et al., 2020; McCauley et al., 2020)
PD	Genetic, expression	Ligand (mtDNA)	Protection from development of motor deficits in a STING-deficient mouse model; STING protein expression in substantia nigra correlates with α-synuclein deposition in patients (Hinkle et al., 2022; Sliter et al., 2018)
NPC	Genetic	Trafficking	STING-deficient mouse model displays improved motor function (Chu et al., 2021)
Huntington’s disease	Expression	Unknown	Elevated p-STING in a mouse model (Sharma et al., 2020)
Ataxia telangiectasia	Pharmacological inhibitor	Ligand (likely gDNA from DNA damage)	Pharmacological inhibition of STING reduces senescence phenotypes and inflammation in brain organoids model (Aguado et al., 2021)
Traumatic brain injury	Genetic, expression	Unknown	Decreased levels of pro-inflammatory cytokines in a STING-deficient mouse model; STING expression and activity upregulated in late trauma human brain samples and a mouse model (Abdullah et al., 2018; Barrett et al., 2020; Sen et al., 2020)
Ischemic brain injury and stroke	Pharmacological inhibitor	Unknown	Pharmacological inhibition of STING reduces brain infarction, neuronal injury, and cognitive deficits in a mouse model (Kong et al., 2022)
Subarachnoid hemorrhage	Pharmacological inhibitor	Unknown	STING inhibition improves edema, neuronal injury, inflammation, and behavioral outcome in a mouse model (Peng et al., 2020)

ALS and FTD are two neurodegenerative disorders that are thought to represent a spectrum of clinical manifestations as well as disease pathology (Root et al., 2021). These two diseases not only share genetic etiologies but also demonstrate the presence of insoluble aggregates of TAR DNA-binding protein 43 kD (TDP-43) in the central nervous system of affected patients (Abramzon et al., 2020; Neumann et al., 2006). TDP-43–mediated neurodegeneration is associated with generation of an inflammatory response, namely cytokines, consistent with an NF-κB response and generation of IFN-I (Egawa et al., 2012; Swarup et al., 2011; Zhao et al., 2015). This inflammation was recently shown to be mediated by cGAS–STING signaling (Yu et al., 2020). Interestingly, this inflammation occurs before the development of neurological defects, suggesting that this response could be a driver of symptomatic onset. The deposition of TDP-43 occurs in the cytoplasm and the mitochondria where it ultimately causes leakage of mtDNA into the cytosol to activate cGAS–STING signaling (Magrané et al., 2014; Wang et al., 2017; Wang et al., 2013). Genetic knockout or inhibition of cGAS–STING signaling abrogates the TDP-43–mediated inflammatory response and protects against disease progression in terms of both survival and behavioral defects in a mouse model of ALS (Yu et al., 2020). Therefore, TDP-43–associated ALS is a clear example of ligand-mediated disease of STING signaling.

The most common genetic defect found in FTD and ALS is a hexanucleotide repeat expansion in *C9orf72,* which leads to decreased protein expression in peripheral blood cells and within the brain (DeJesus-Hernandez et al., 2011; Renton et al., 2011; Belzil et al., 2013). Loss of C9orf72 in myeloid cells leads to impaired degradation of STING by the lysosome leading to hyperactive IFN-I production (McCauley et al., 2020). This suggests that the hyperinflammatory response seen in ALS and FTD may also be a result of a trafficking-mediated enhancement of STING signaling. Further, data from recent years has strengthened the association between lysosomal dysfunction and pathogenesis of FTD and ALS (Root et al., 2021). Impaired lysosomal function, autophagy and vesicle trafficking are observed in postmortem tissue from these patients (Lie and Nixon, 2019; Farg et al., 2014). In fact, several genetic mutations in genes attributed to FTD, including *C9orf72*, *GRN*, *MAPT*, *TARDBP*, *TMEM106B*, *VCP*, *CTSF*, and *SQSTM1* among others, encode lysosomal proteins that affect lysosomal acidification and enzymatic activity (Root et al., 2021). It will be interesting to see whether STING signaling has a broader impact on ALS and FTD pathogenesis than currently appreciated. Lysosome dysfunction in some cases of FTD and ALS also leads to mitochondrial damage. Thus, a singular disease could display components of both ligand-mediated and trafficking-mediated activation of STING signaling. The contributions of each to symptomatic development and pathological progression must be carefully dissected.

PD is a progressive neurodegenerative disorder mainly affecting movement. Multiple mechanisms of STING activation have been proposed for PD. In a mouse model of PD (induced by α-synuclein injection), increased TBK1 activation and accumulation of DNA damage were observed, providing a possible ligand for cGAS–STING activation (Hinkle et al., 2022). Accordingly, genetic knockout of STING in this mouse model protected mice from development of motor deficits, α-synuclein accumulation, and dopaminergic neuron loss which are hallmarks of PD. Interestingly, STING protein levels are upregulated in the substantia nigra of PD patients, and this increase correlates with α-synuclein accumulation. In other mouse models of PD (Parkin-, PINK1-, or VPS13C-deficient mice) mitochondrial damage and mtDNA activate cGAS–STING–IFN signaling and contribute to loss of dopaminergic neurons (Sliter et al., 2018; Hancock-Cerutti et al., 2022). Many other PD-associated genes encode endolysosomal proteins (Shachar et al., 2011), raising the interesting possibility that mutations in these proteins could impair STING degradation, thus enhancing STING-mediated immune signaling.

The discovery of COPA syndrome as a STING-mediated disease leads to the question of whether other diseases associated with COPI dysfunction are driven in part by constitutive STING activation. Numerous neurodegenerative diseases are linked to COPI dysfunction including distal hereditary motor neuropathies and Charcot-Marie-Tooth neuropathy (Mendoza-Ferreira et al., 2020). The contribution of STING signaling to the development of these disease pathologies is unexplored.

### Therapeutic opportunity

STING agonists have been actively developed for cancer immunotherapy. STING antagonist development effort is very limited largely because relatively few diseases are associated with overt STING signaling and many of them are rare inborn error diseases. A few small-molecule inhibitors of STING have been developed so far (Haag et al., 2018; Decout et al., 2021). However, the landscape is quickly changing. Thanks to recent discoveries of STING signaling in driving neurodegenerative disease, we should see better and more specific STING inhibitors in the coming years.

Another therapeutic opportunity is to develop a new class of STING agonists based on trafficking-mediated activation. Current STING agonists all target the ligand-binding pocket of the STING protein. These agonists stimulate an acute and potent response (“fast and furious”) but are often associated with tissue toxicities that limit their efficacy in clinical trials. As an alternative, we believe trafficking-mediated activation could produce chronic low-level STING signaling (“slow and steady”) that eventually reaches the same clinical benefit with fewer side effects. As a proof of concept, we showed that STING trafficking interruption (e.g., *Gcc2*^*−/−*^ and *Rab14*^*−/−*^) generates antitumor immunity that is functionally on par with STING agonists (T cell–dependent and IFN-dependent). Therefore, future compounds that selectively interrupt post-Golgi STING trafficking may represent the next generation of STING activators for cancer immunotherapy.
